# Twisting and ignoring facts on hydroxyethyl starch is not very helpful

**DOI:** 10.1186/1757-7241-21-85

**Published:** 2013-12-09

**Authors:** Daniel Chappell, Matthias Jacob

**Affiliations:** 1 Department of Anaesthesiology, University Hospital of Munich, Nußbaumstrasse 20, 80336 Munich, Germany

**Keywords:** Colloids, Crystalloids, Fluid therapy, Hydroxyethyl starch, Sepsis

## Abstract

Large randomized trials on critically ill patients have related the use of hydroxyethyl starch (HES) to negative outcomes. In a recent comment we explained in detail why, from our point of view, transferring the results of VISEP, 6S and CHEST into daily ICU practice is as difficult as their extrapolation to perioperative treatment. Haase, Müller and Perner lately challenged this analysis. However, after having carefully read their letter to the editor we are happy to demonstrate that all points we made were absolutely correct. We agree with Haase et al. that a debate on HES safety is important, but has to be based on facts. The difference might be that we like to thoroughly discuss all of them, including the main one: VISEP, 6S and CHEST do not capture the initial stabilization of their hemodynamically instable patients. The vast majority, including those patients later assigned to the “crystalloid” groups, had been stabilized with colloids before study onset. This is not a big problem, but has to be discussed carefully and honestly to prevent the data from being misinterpreted by users and official authorities.

## 

“Facts do not cease to exist because they are ignored”. *Aldous Huxley 1929.*

We are grateful to Drs. Haase, Müller and Perner for their interest in our thoughts and happy that we agree in their major point: discussions on hydroxyethyl starch (HES) are important, but have to be based on ***facts*** [1]. Unfortunately, their reply might arouse the suspicion to the reader that our comment [2] was not accurate. This is certainly not the case, all numbers and statements were correct. We are happy to provide some help concerning ***the facts*** in this discussion.

First, Haase et al. ignore ***the fact*** that fresh frozen plasma (FFP) contains albumin and other macromolecules and, therefore, is a colloid. Taking this into account, in 6S [3] the majority of the patients in the crystalloid group received ***in fact*** up to 1 liter of colloids (HES, albumin and/or FFP) for initial stabilization before the trial started. After that, 1/3 of this “crystalloid” group additionally received a colloid during the interventional period. Therefore, 6S is ***in fact*** not a trial purely comparing crystalloid vs. colloid, but colloid in a usual vs. colloid in an unusual way. As to be expected, the usual way - infusing a considerable amount of colloid in the first hours of severe sepsis before switching to a crystalloidal-based maintenance protocol - led to a significantly better survival. We (and many others worldwide) cannot understand how this can lead to the conclusion that one of these applied colloids should be generally abandoned. The bottom line of Haase et al. does not reflect their results, as over 75% of all patients in their trial received HES.

Haase et al. state that neither they nor we could know what the doctors were doing in detail during the trial, especially what their decision to infuse the study fluid or not was based on in the individual case. We are afraid this is insufficient to serve as a reliable reference for worldwide future behavior. How should anybody recapture what happened to the patients if even the authors can not? How can the authors draw their incomprehensible conclusions without knowing what exactly happened? From studies which want to change our behavior around hemodynamic optimization a protocol including basic and extended hemodynamical monitoring, a careful documentation of the need of vasopressors/inotropic agents and criteria for implementing, e.g., renal replacement therapy is absolutely necessary.

Nevertheless, we agree that 6S indicates how well the participating doctors were trained: They infused colloids during the crucial initial resuscitation phase to resuscitate their patients.

The next point our comment criticized was ***the fact*** that the duration of ICU stay was not reported. According to the opinion of Haase et al., the aspect “alive and out of hospital” chosen instead would provide more information, as it might be “less affected by survival bias”. However, ***a fact*** is that the patients received trial treatment during their entire ICU stay. Therefore, length of ICU stay is the only number which can tell us how long patients were treated with the respective drug. Despite being a nice additional information, the provided alternative is no scientifically adequate replacement.

It is an interesting explanation for ***the fact*** that over 1/3 of the patients received a *contraindicated* drug that the steering committee, scientific advisors and Medicine Agencies approved the protocol. We are convinced that approval does not release from medical responsibility and, therefore, does not justify to ignore ***the fact*** that renal failure was already a contraindication for HES (stated e.g. by the American FDA [4]) before 6S was initiated and it still is one today. Even if the authors believe this inclusion to be ethical, we would at least expect a subgroup analysis excluding these patients.

We agree with Haase et al. that it takes time to identify “severe sepsis” and to fulfill all the regulatory requirements. Accordingly, an earlier inclusion of septic patients into double-blinded prospective studies is hardly possible. However, this does not justify a discussion ignoring their major weakness: ***the fact*** that colloids (predominantly *HES)* were used in the vast majority of patients to reach the hemodynamical target criteria of the Surviving Sepsis Campaign [5]*before* study treatment started, also in those of the later “crystalloid” groups. Moreover, it is important to discuss ***the fact*** that in 1/3 of the “crystalloid” group colloids were also infused *during* the trial. Again, a subgroup analysis would have been quite informative here.

In this context it is interesting that the recently published CRISTAL-study [6], a randomized open label trial on 2,857 patients in hypovolemic shock for various reasons (sepsis, trauma, or without sepsis or trauma), included the crucial first 6 hours of resuscitation [7] into the treatment protocol, showing completely different results. Treating the patient with any kind of colloid (starch, gelatin or albumin) compared to any kind of crystalloid, CRISTAL showed an improved 90-day survival, fewer days of mechanical ventilation and less need of vasopressors in the colloid group. No differences in the incidence of organ failure or renal replacement therapy were detected [6]. The strategy of CRISTAL to combine different patient collectives and different colloids into one analysis may not be ideal. However, this study shows, for the first time, that an *indication-based* use of colloids - by the way: the majority of “colloid”-patients received a 3rd generation HES - might be advantageous to patients. In our opinion this is at least as helpful as demonstrating a probably artificial problem related to the continuous use of HES in large amounts *after* stabilization, i.e., the use of a potent drug in a way that is not well established in clinical practice.

Last but not least Haase et al. declare that “safer ways of using HES in critically ill patients have not yet been identified”. This is astonishing. First, in their own trial early hemodynamical stabilization was performed with HES and other colloids. This obviously was the basis of outcome success even in their septic patients who received crystalloids later on. A pure crystalloidal therapy from the very beginning of stabilization was not evaluated in any group of 6S. Therefore, there is no scientific basis of the author’s recommendation to principally rely on such a strategy in the future. Beyond that, there are clear hints in literature showing that a perioperative goal-directed therapy using HES during major surgery reduced postoperative complication rates, hospital stay and morbidity.

Nevertheless, we agree with Haase et al. that in 2013 no patient should be treated with HES *following* initial stabilization, especially in sepsis. As ***a fact***, this is what 6S actually shows.

We are grateful to each scientist who runs a large RCT addressing fluid and volume handling. However, twisting facts, ignoring contraindications, hiding vital information and over-interpreting results is not helpful to advance the scientific discussion. Rather, it is dangerous for the patients. It is not surprising that, after having carefully reconsidered also the latest data with the help of independent experts the Pharmacovigilance Risk Assessment Committe (PRAC) of the European Medicines Agency (EMA) revised its first decision from June markedly. This initiated the Coordination Group for Mutual Recognition and Decentralized Procedures - Human (CMDh) in October to vote for maintaining hydroxyethyl starches on the market for treatment of hypovolemia due to acute bleeding. Very soon, the European commission will make a final decision and it is most likely that it will be close to the CMDh-recommendation. The planned restrictions for septic, critically ill and burned patients are sensible and comprehensible. However, until today there are no high quality data supporting that for the initial stabilization phase in shock for any reason.
